# Single dose rVSVΔG-JUNVGP vaccine protects guinea pigs against lethal Junin virus challenge

**DOI:** 10.1038/s41541-021-00361-0

**Published:** 2021-08-09

**Authors:** Teresa E. Sorvillo, Robert W. Cross, Dylan M. Johnson, Natalie S. Dobias, Karla A. Fenton, Chad E. Mire, Thomas W. Geisbert

**Affiliations:** 1 Galveston National Laboratory, University of Texas Medical Branch, Galveston, TX USA; 2 Department of Microbiology & Immunology, University of Texas Medical Branch, Galveston, TX USA

**Keywords:** Arenaviruses, Viral infection, Experimental models of disease

## Abstract

Junin virus (JUNV) is a pathogen of biodefense importance due to its potential for aerosol transmission and mortality rates reaching 30%. Currently, there are no JUNV vaccines licensed by the United States Food and Drug Administration (FDA) for at-risk individuals. A vaccine based on recombinant vesicular stomatitis virus (rVSV) has been effectively used to prevent Ebola virus disease in humans. Here, we evaluated the protective efficacy of a rVSV expressing the JUNV glycoprotein (rVSVΔG-JUNVGP) in a guinea pig model of lethal JUNV disease. Two groups of guinea pigs, one prime and one prime-boost, were vaccinated with rVSVΔG-JUNVGP; six control animals remained unvaccinated. Survival for prime and prime-boost vaccinated animals was 100% while the challenge virus was uniformly lethal in all control animals. Animals in both vaccine groups developed robust, high avidity IgG antibody titers post-vaccination as well as detectable neutralizing antibodies while control animals failed to develop detectable antibody responses. This study demonstrates for the first time that rVSV expressing the JUNV GP fully protects guinea pigs from lethal JUNV challenge with a single injection vaccine.

## Introduction

Junin virus (JUNV), the causative agent of Argentine hemorrhagic fever (AHF), is a significant human pathogen and member of the family *Arenaviridae*^1^. It is categorized by the National Institute of Allergy and Infectious Diseases (NIAID) as a Category A priority pathogen due to its virulence and potential for aerosol transmission, making it a prominent public health and biodefense concern^2^. JUNV is endemic to the Pampas farming region of Argentina where an estimated 5 million persons are at-risk of infection^3,4^. The virus was first described in 1953 and the incidence of AHF averaged between 500 and 3500 cases per year until 1991 when administration of a live-attenuated JUNV vaccine to high risk populations in the endemic area was initiated^3^. Despite the vaccine’s effectiveness at decreasing JUNV incidence, there are still several AHF cases reported annually in the endemic area^5^. JUNV infection typically occurs via inhalational exposure to the excrement or fluids of infected rodents. AHF begins with a flu-like illness 4–21 days after JUNV exposure after which a severe, late-stage disease which is characterized by either neurologic or hemorrhagic symptoms may develop. Overall, mortality rates for persons with symptomatic AHF are as high as 30%^1,4,6^.

Therapeutic countermeasures available for the treatment of AHF are limited. The antiviral drug ribavirin, although not licensed to treat JUNV, has been shown to delay time-to-death in nonhuman primates (NHP) but provides minimal protection unless administered prophylactically^7,8^. Clinical trials of ribavirin indicate a similar lack of efficacy in human patients, likely due to the absence of symptom specificity during the first week of infection resulting in delayed diagnosis and treatment^4,7,9,10^. Convalescent plasma from JUNV survivors has been shown to be effective as a therapeutic. A double-blind placebo-controlled study determined that administration of immune plasma reduced overall case-fatality from 16.5 to 1.1%; however, there are inherent difficulties with maintaining and ensuring the safety of plasma stocks^4,7,11^. Importantly, the protective efficacy of immune plasma has been shown to correlate with neutralizing antibody titer, a finding that has prompted additional research to focus on the development of monoclonal antibody-based therapies, although none are yet licensed for use in humans^12,13^.

A live-attenuated JUNV strain named Candid #1 is the only available vaccine for the prevention of AHF in Argentina and studies have demonstrated that the vaccine is both immunogenic and effective. An average of 91.1% of vaccinated persons seroconvert within 5 months of a single intramuscular vaccination and over a 9-year period the vaccine effectiveness was determined to be 98.1%^14–16^. Although effective in Argentina, Candid #1 has several significant limitations which preclude it from receiving Food and Drug Administration (FDA) approval in the United States. Specifically, Candid #1 retains a low level of neurotropism. A study in rhesus macaques vaccinated intramuscularly with Candid #1 resulted in neuronal lesions that were consistent with wild type JUNV infection in 4 out of 24 animals^3^. Candid #1 also has an unstable attenuated phenotype, with a single in vivo passage yielding isolates up to 100-fold more neurovirulent than the parental attenuated virus^3^. Further, the attenuated phenotype is believed to rely heavily on a single amino acid substitution (F427I) in the glycoprotein’s (GP) transmembrane domain^17^. Notably, adverse events associated with Candid #1 have been reported to be as high as 29–35% and include low platelet count, vomiting, and fever^15^. Due to these limitations, an alternative JUNV vaccine that can overcome these hurdles for FDA licensure should be explored. One such alternative could be the use of the recombinant vesicular stomatitis virus (rVSV) vaccine vector system.

The VSV reverse genetics system, first developed by Lawson et al.^18^, consists of a Bluescript plasmid containing the full length VSV genome which encodes the following viral proteins: nucleoprotein (N), phosphoprotein (P), matrix protein (M), glycoprotein (G), and RNA-dependent RNA polymerase (L). Inserting foreign glycoprotein sequences into the VSV reverse genetics system and recovering rVSVs has been procedurally well-established with a significant history of success^19–23^. In addition, a rVSV-based vaccine has been effective in providing protection against *Zaire ebolavirus* in humans. Notably, a Phase III clinical trial of the rVSV *Zaire ebolavirus* vaccine in Guinea demonstrated 100% vaccine efficacy and was subsequently administered to over 200,000 people in the Democratic Republic of the Congo to combat a 2018 outbreak of the virus; the vaccine has since received FDA approval (2019)^24^. Studies have also demonstrated evidence of vaccine durability via strong IgG antibody persistence in human vaccinees for at least 2 years. Further, a rVSV expressing the glycoprotein precursor (GPC) gene of Lassa virus (LASV), a prominent Old World arenavirus, has been shown to be fully protective against lethal LASV challenge in NHPs, indicating the same strategy may be promising for the development of a JUNV vaccine^25^.

The JUNV GP is a potent immunogen which elicits protective neutralizing antibody responses. It is a trimeric protein expressed on the virion surface that is used for JUNV entry and comprised of the major subunits: G1, G2, and stable signal peptide (SSP)^26^. Studies have shown that antibodies directed exclusively against the JUNV GP produce robust neutralization titers and are sufficient to fully protect guinea pigs from lethal JUNV challenge, suggesting that the GP would be a prudent antigen choice for JUNV vaccine development^27^.

Here, we present data on the recovery of a rVSV expressing the JUNV GP (rVSVΔG-JUNVGP) and characterization of this vaccine vector. In addition, we assessed rVSVΔG-JUNVGP as a vaccine in a lethal JUNV guinea pig challenge model.

## Results

### rVSVΔG-JUNVGP characterization

**Fig. 1:**
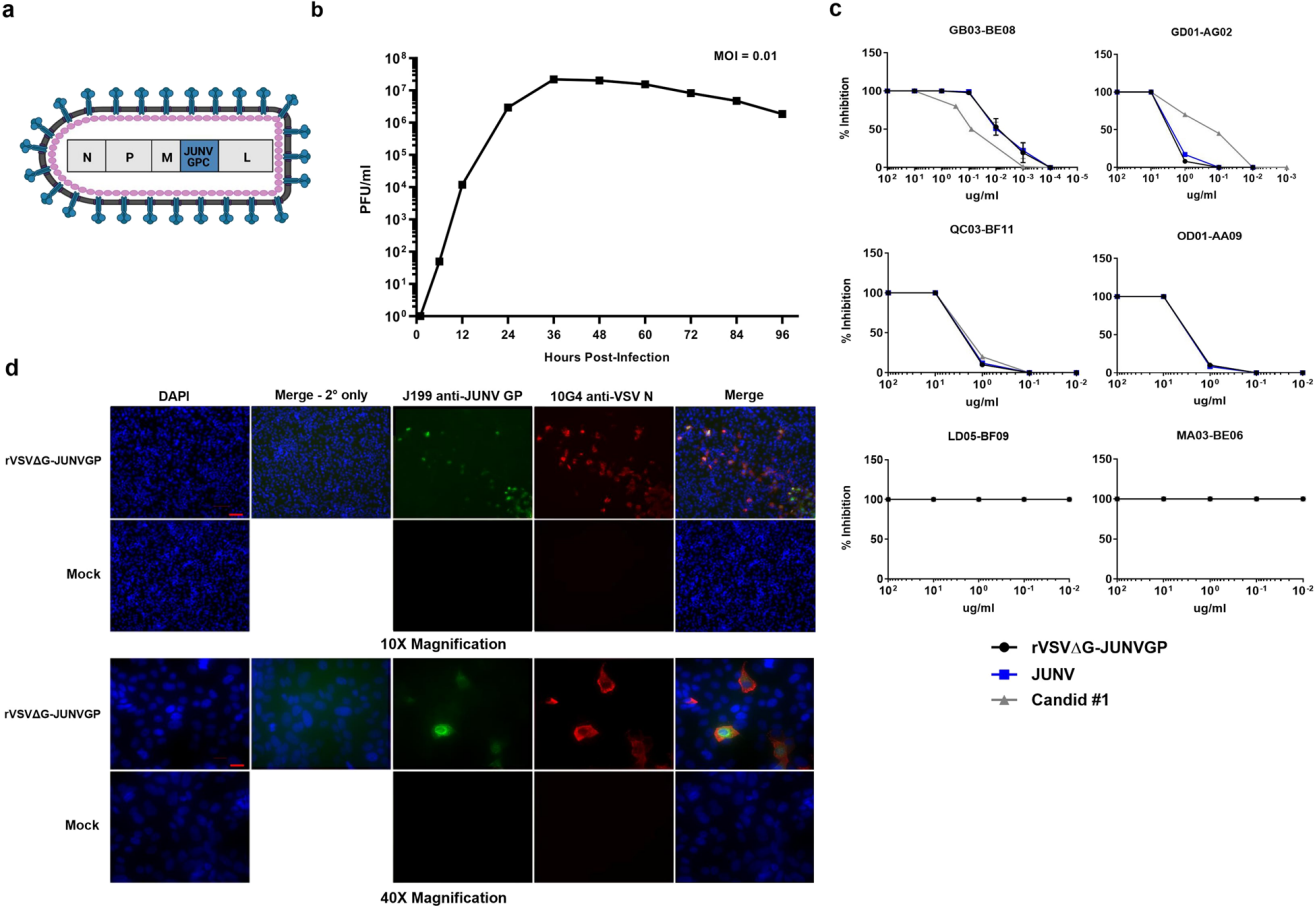
rVSVΔG-JUNVGP vaccine design and characterization. **a** Representative image of the rVSVΔG-JUNVGP virion, elongated and bullet-shaped, expressing JUNV GP on the surface. The genome contains: VSV nucleoprotein (N), VSV phosphoprotein (P), VSV matrix protein (M), JUNV GPC, VSV polymerase (L). **b** Growth kinetics of rVSVΔG-JUNVGP in Vero76 cells inoculated at a MOI of 0.01. rVSVΔG-JUNVGP supernatants were collected every 12 h from 0 to 96 h with peak viral titer at 36 h. Viral titers were evaluated via plaque assay (25 PFU/ml limit of detection). **c** Plaque reduction neutralization tests of rVSVΔG-JUNVGP, wild-type JUNV Espindola, and Candid #1 viruses with six distinct JUNV monoclonal antibodies (described by^28^). Antibodies GB03-BE08, OD01-AA09, GD01-AG02, and QC03-BF11 are JUNV neutralizing antibodies, while LD05-BF09 and MA03-BE06 are non-neutralizing and target the JUNV GP and JUNV NP, respectively. Error bars representative of standard deviation of the mean of replicate measures. **d** Immunofluorescence assay (IFA) of rVSVΔG-JUNVGP infected Vero76 cells. Co-expression of VSV N (red) and JUNV GP (green) protein is observable in the same cells. Cell nuclei are visible (blue) via DAPI counterstain. Scale bars for 10x images are 100 micrometers and 40× image are 20 μm.

We were successful in recovering rVSVΔG-JUNVGP (Fig. 1a) and generating a growth curve to demonstrate replication competence in Vero76 cells where peak viral titer occurred 36 h post-infection (PI) (Fig. 1b). Immunostaining for VSV N and JUNV GP in Vero76 cells resulted in observable co-expression of VSV N and JUNV GP protein in the same cells (Fig. 1d). Sequencing was also performed on plaque purified virus seed stocks which revealed a single amino acid substitution (I101F) in G1. While it is possible that a single amino acid mutation could impact virus phenotype and/or vaccine immunogenicity, our decision to proceed with in vivo experiments was based on several factors including the similarity of isoleucine and phenylalanine in having hydrophobic side chains of like size. In addition, we performed neutralization assays of rVSVΔG-JUNVGP with several well-characterized and protective JUNV monoclonal antibodies (described by^28^), which demonstrated that our VSV construct (I101F) was effectively neutralized in a manner consistent with wild type JUNV Espindola (Fig. 1c).

### In vivo vaccine efficacy

**Fig. 2:**
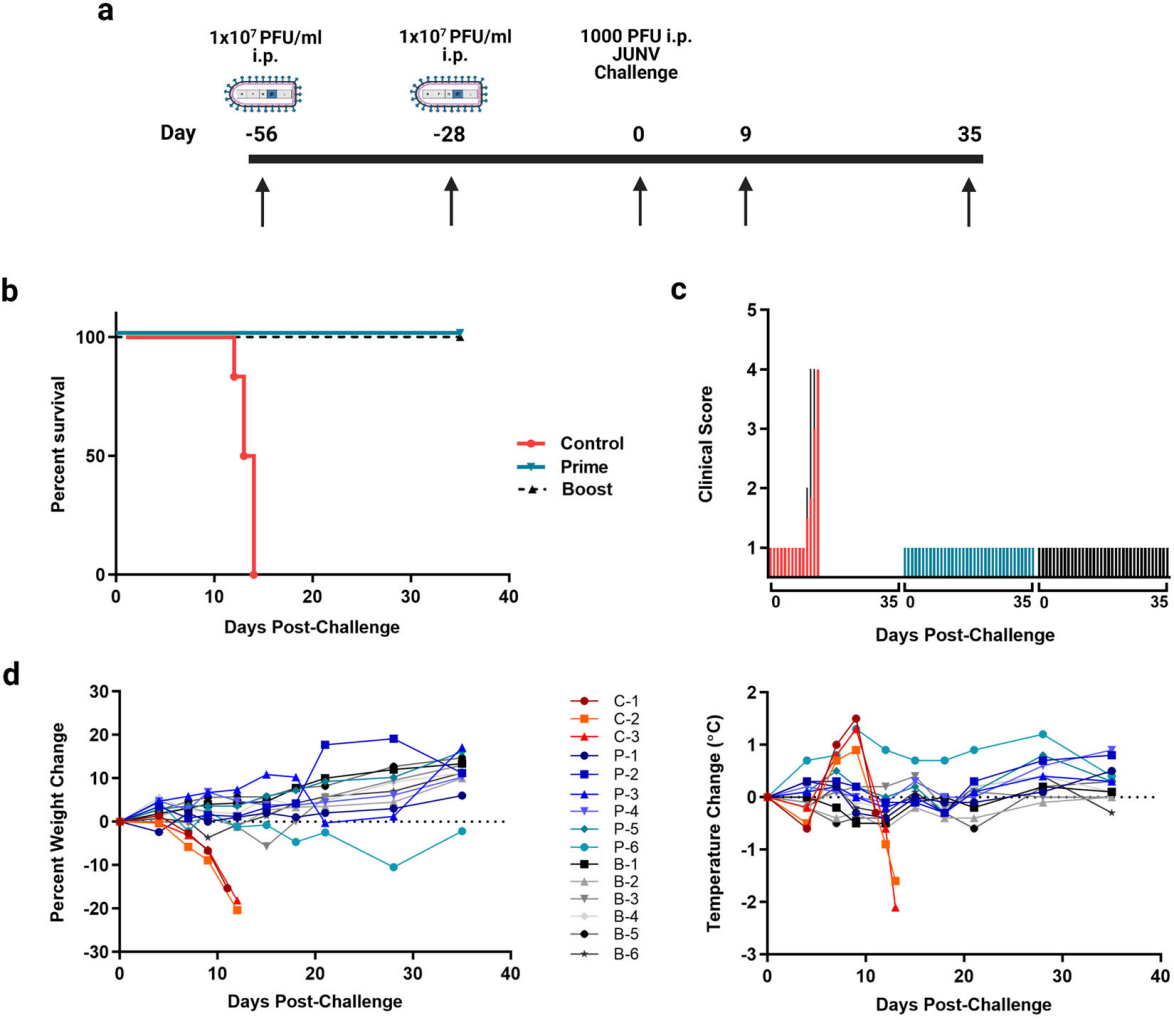
rVSVΔG-JUNVGP vaccination and protective efficacy in JUNV challenged guinea pigs. **a** Timeline for rVSVΔG-JUNVGP vaccination study in guinea pigs. Prime and prime-boost cohorts were vaccinated on day −56 with 1e7 PFU of rVSVΔG-JUNVGP. The prime-boost cohort was vaccinated again on day −28 with 1e7 PFU of rVSVΔG-JUNVGP. All animals were challenged with 1000 PFU of JUNV Romero on day 0. Arrows indicate dates of plasma collection: −56, −28, 0, 9, 35 or terminal. **b** Kaplan–Meier survival curve for prime, prime-boost, and control guinea pig groups. **c** Average clinical scores on days 0–35 for prime, prime-boost, and control animals. Scores were characterized as the following: Normal (1), Rough (2), Sick (3), Paralysis and/or Euthanize (4). **d** Guinea pig weight and temperature changes over time. Baseline measurements were taken on day 0. Additional measurements were taken every 3 days through day 21, day 28 and day 35 or terminal. For all graphs, control animals are represented in red, prime animals in blue, and prime-boost animals in black/gray.

To assess the protective efficacy of rVSVΔG-JUNVGP, we utilized a lethal JUNV guinea pig model^13^. Two groups of six guinea pigs each received a prime dose (1 × 10^7^ plaque forming units (PFU) administered intraperitoneally (i.p.)) of rVSVΔG-JUNVGP fifty-six days before lethal JUNV challenge. One group received an identical boost dose twenty-eight days before challenge (Fig. 2a). All control animals remained unvaccinated. In both vaccinated cohorts the vaccination regimens proved to be 100% efficacious while there was uniform lethality in the control cohort (Fig. 2b). It is important to emphasize that the JUNV Romero guinea pig challenge model used in this study reliably results in uniform lethality (1000 PFU i.p. challenge dose) and the control animals described here succumbed to disease as previously described by Zeitlin et al.^13^.

Post JUNV-challenge, all animals were followed for changes in temperature, weight, and clinical score. Temperatures became elevated over baseline beginning on day 7 (+0.7 °C to +1.0 °C) and peaking on day 9 (+0.9° to +1.5 °C) for control animals (Fig. 2d). Temperatures remained near baseline values in all vaccinated animals for the duration of the study with the exception of a single animal (P-6). Elevated temperature in this animal began on day 4 (+0.7 °C) and peaked on day 9 (+1.3 °C) but remained higher than average until day 35 when it returned to baseline (Fig. 2d). Control animals were found to have detectable weight loss starting on day 7 which continued until euthanasia (Fig. 2d). Conversely, all vaccinated animals gained weight consistently for the duration of the study post-challenge. A single outlier, again animal P-6, began to lose weight on day 7 which continued until day 28 (−10%); however, the animal returned to baseline weight by day 35 (Fig. 2d). Overall assessment of clinical score data indicated that clinical signs of disease were not observed in any of the vaccinated animals, including animal P-6 (Fig. 2c).

**Fig. 3:**
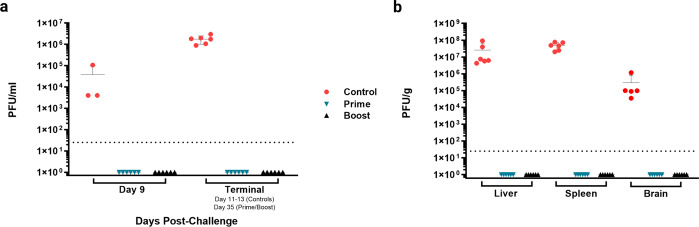
JUNV titer in plasma and tissue after rVSVΔG-JUNVGP vaccination and JUNV challenge. **a** Plasma titers (PFU/ml) for prime, prime-boost, and control groups on days 9 and 35. **b** Terminal liver, spleen, and brain titers (PFU/g) for prime, prime-boost, and control groups. All control animals are represented in red, prime animals in blue, and prime-boost animals in black/gray.

Notably, none of the vaccinated animals had detectable viremia on days 9 or 35, including animal P-6, while control animals had comparable titers of circulating virus on day 9 and at euthanasia (Fig. 3a). The same pattern was seen for virus load in tissue. Control animals had equivalent infectious virus isolated from the liver, spleen, and brain at terminal time points while no detectable JUNV was found in the tissues of vaccinated animals at study end (Day 35) (Fig. 3b).

**Fig. 4:**
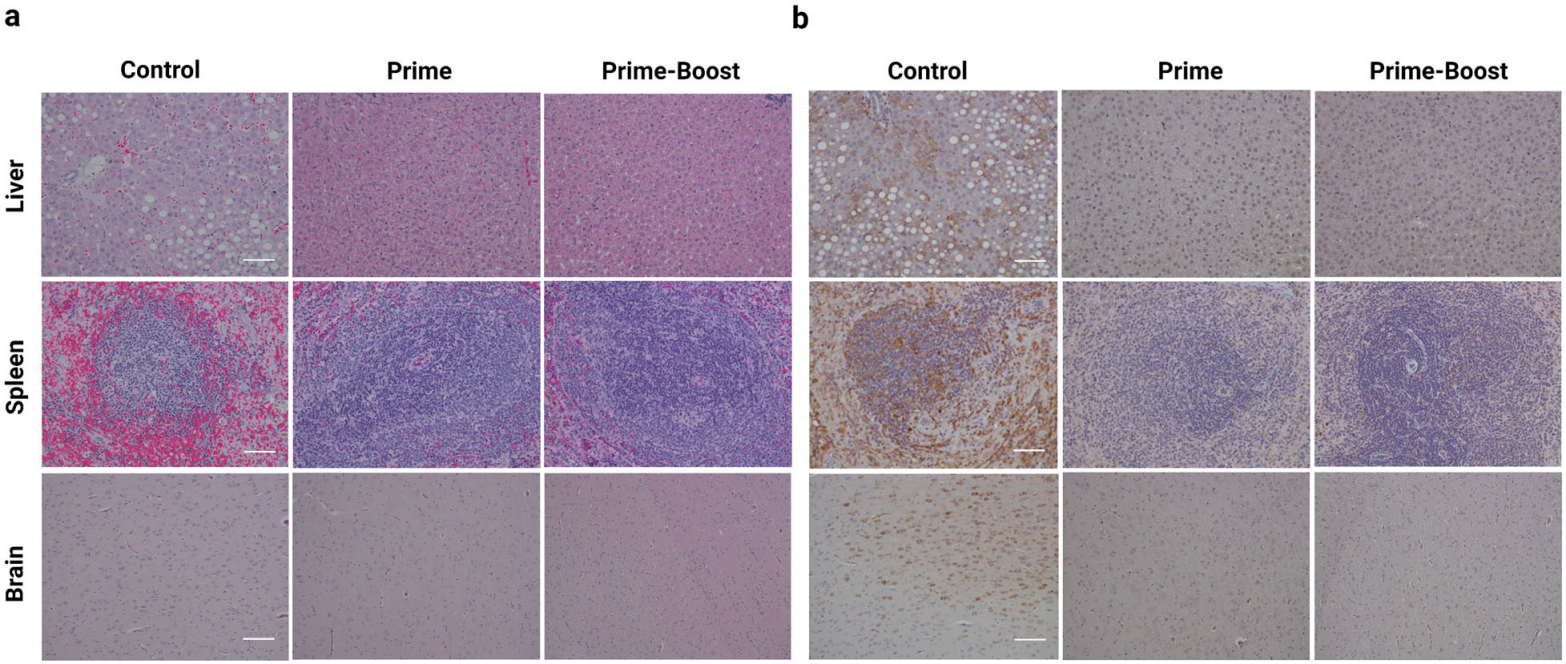
Histological analyses of liver, spleen, and brain after rVSVΔG-JUNVGP vaccination and JUNV challenge. Representative images from control, prime, and prime-boost vaccinated animals. **a** H&E: tissue stained with hematoxylin and eosin. Control animals have observable hepatocyte vacuolation (liver), germinal center degeneration characterized by lymphoid depletion and hemorrhage (spleen), and gliosis (brain). No significant lesions are observable in the liver, spleen, or brain of prime or prime-boost vaccinated animals. **b** IHC: tissue stained with JUNV-specific antibody. Control animals have observable immunolabeling of hepatocytes (liver), germinal centers (spleen), and neurons (brain). No detectable immunolabeling in the liver, spleen, or brain of prime or prime-boost vaccinated animals. Brain images are 10x and spleen/liver are 20x magnification; scale bar 100 μm (brain) and 20 μm (spleen/liver).

The tissues of control animals were observed to have characteristic histopathologic changes from JUNV infection including diffuse hepatocellular vacuolar degeneration in the liver, and germinal center degeneration, lymphoid depletion, and hemorrhage in the spleen (Fig. 4). Lesions were not observed in the liver, spleen, or brain of vaccinated animals via histology (Fig. 4). JUNV-specific antigen labeling was detected in all three tissues of control animals, but notably, viral antigen was not detected in the tissues of vaccinated animals (Fig. 4), including the brain.

### rVSVΔG-JUNVGP-induced antibody responses

**Fig. 5:**
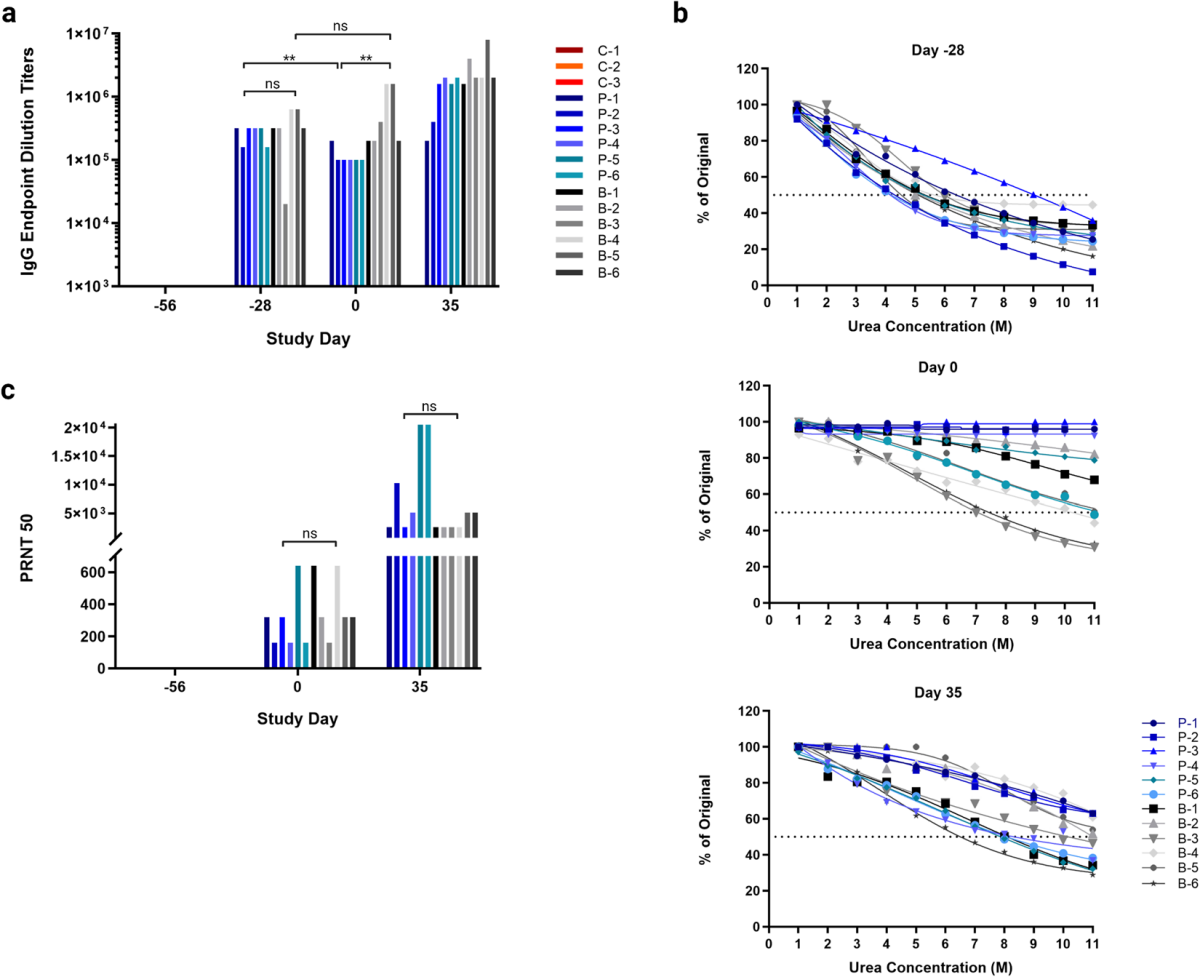
Characterization of humoral immunity after rVSVΔG-JUNVGP vaccination and JUNV challenge. **a** IgG reciprocal endpoint dilution titers for vaccinated animals on days −56, −28, 0, and 35. **b** IgG antibody avidity on days −28, 0, and 35. Data reported represent the dissociation of antibody/antigen binding with increasing concentrations of urea (1 M through 11 M). **c** Neutralization titers for vaccinated animals on days −56, 0, and 35. For all graphs, control animals are represented in red, prime animals in blue, and prime-boost animals in black/gray. All statistical notations correspond with the following *p* values: *p < 0.05, **p < 0.01, ***p < 0.001.

The survival and virus load data revealed that both vaccination regimens were 100% efficacious against JUNV challenge. To evaluate if there were any differences between the immune response to either vaccine regimen, JUNV GP-specific IgG antibody titers were assessed for all animals on days −56, −28, 0, and day 35 or terminal. All vaccinated animals seroconverted, developing uniformly robust IgG antibody titers by day −28; no statistically significant difference in titer was detected between prime and prime-boost animals (Fig. 5a). On day 0 IgG titers from animals that received a single vaccination were significantly lower than on day −28; however, these animals were able to maintain robust IgG titers for at least 4 weeks before being challenged (Fig. 5a). IgG titers in animals receiving prime-boost vaccinations increased overall from day −28 to day 0; however, this increase was not statistically significant (Fig. 5a). There was a statistically significant difference in IgG titer between the prime and prime-boost animals at JUNV challenge on day 0; however, at the time of challenge all animals had high JUNV GP-specific IgG titers in excess of 1e5 (reciprocal dilution). Within the confines of our study timeline, circulating IgG titers were highest for both groups on day 35 post-challenge (Fig. 5a). Control animals failed to develop detectable JUNV-specific IgG antibody during the study.

In addition to IgG titer we also assessed IgG antibody quality via an ELISA-based avidity assay. We found that by day −28 all vaccinated animals had developed IgG antibodies with similar avidity profiles, where 50% dissociation occurred with exposure to 4 M–6 M urea with the exception of a single outlier, animal P-3 (9 M urea) (Fig. 5b). Importantly, overall avidity was higher in all animals by day 0, suggesting the presence of B-cell affinity maturation over time (Fig. 5b). Interestingly, although overall avidity was higher on day 0, animals in the prime group were found to have significantly higher avidity on average than animals in the prime-boost group (Fig. 5b). On day 35, animals in both prime and prime-boost groups were found to have lower antibody avidity overall than on day 0.

Finally, we assessed the neutralizing antibody responses resulting from rVSVΔG-JUNVGP vaccination. All vaccinated animals developed detectable neutralizing antibodies by day 0 with PRNT_50_ values ranging from 160–640 (reciprocal dilution) (Fig. 5c). Neutralization titers were found to be higher overall in the prime-boost versus prime animals on day 0; however, this difference was not statistically significant (Fig. 5c).

## Discussion

Vaccine and therapeutic options are limited for the prevention and treatment of AHF making the development of countermeasures against JUNV an important public health and biodefense priority. rVSV-based vaccines have demonstrated protective efficacy against hemorrhagic fever viruses in many relevant animal models including mice, hamsters, guinea pigs, and NHPs, as well as in a recent clinical trial in human patients^19–21,23,24^. In this study we demonstrated that rVSV expressing the JUNV GP protects guinea pigs from lethal JUNV challenge with a single vaccine injection.

Significantly, rVSVΔG-JUNVGP-vaccinated animals failed to develop signs of clinical disease post-challenge. Weight loss and elevated temperatures were detected in a single animal; however, no circulating infectious virus could be detected on day 9 or 35 in any vaccinated animal. In addition, no virus, viral antigen, or histopathologic changes were found in the liver, spleen, or brain of surviving animals. These findings regarding the brain are particularly important because rodent models of JUNV occasionally present with signs of late neurologic disease which is thought to be the result of viral recrudescence from persistence in the brain^29,30^. They also indicate that rVSVΔG-JUNVGP may successfully circumvent the issues of neurotropism associated with Candid #1 vaccination. However, additional studies should be performed to verify the accuracy of these findings beyond the 35-day study end point.

Adaptive immune responses have previously been demonstrated as essential for JUNV survival in both animals and humans, particularly antibody responses directed against the viral GP which can both neutralize the virus and facilitate Fc-mediated effector functions^12,27,31,32^. Further experiments will need to be performed to understand vaccine correlates of protection; however, we found that all vaccinated animals mounted high avidity, high titer IgG antibody responses and developed neutralizing antibodies by the day of challenge, all of which may contribute to protection. Importantly, all vaccinated animals developed robust IgG titers after the initial prime vaccination and only three animals had a notable increase in IgG titer after boost, suggesting a boost may not be necessary to generate high titer IgG antibody responses.

Similarly, we found that animals receiving only a single vaccine injection had statistically higher antibody avidity at the time of challenge compared with prime-boost animals, suggesting a boost may also not be necessary for the generation of high avidity antibody responses. While the scope of this work was not to investigate the mechanism behind this avidity difference, we would suggest that a secondary, boost vaccination serves to drive additional primary waves of immune activation while also driving affinity maturation in existing B-cell populations, the sum of which produced lower overall avidity in our prime-boost animals^33^. Although the avidity on day 0 was lower in prime-boost animals, we have shown that overall IgG titers were higher. It is possible that the binding strength of high affinity antibodies in the population were diluted by the additional primary immune responses/activation induced by the boost. This would also explain the overall decrease in antibody avidity in both cohorts after JUNV challenge (day 35). Further studies are required to understand the avidity differences between vaccine regimens but overall, our data suggest that a boost may not be necessary and support the idea of moving forward with rVSVΔG-JUNVGP as a single injection vaccine.

Here, we report for the first time, a rVSV-based vaccine against JUNV which demonstrates 100% efficacy against lethal JUNV challenge. We have shown a single injection vaccine regimen can induce a robust and protective immune response, supporting further exploration of rVSVΔG-JUNVGP as a possible candidate vaccine for both public health and biodefense purposes.

## Methods

### Viruses and cell lines

Vero76 cells (American Type Culture Collection- ATCC) were maintained in Eagle’s minimum essential medium (EMEM), 10% fetal bovine serum (FBS), 1% penicillin/streptomycin (P/S), and 1% GlutaMAX. Baby hamster kidney cells (BHK) (Michael Whitt, University of Tennessee Health Science Center) were maintained in Dulbecco’s Modified Eagle’s Medium (DMEM), 5% FBS, 1% P/S, and 1% GlutaMAX. JUNV seed stocks were obtained from Thomas Ksiazek, University of Texas Medical Branch (UTMB). JUNV strain Espindola (P3790) was passaged twice in VeroE6 cells and once in Vero76 cells with prior passage history in mice (2×). JUNV strain Romero (P3235) was passaged three times in Vero E6 cells with prior passage history in mice (2×) and medical research council (MRC) cells (×2). All experiments conducted with JUNV were performed in biosafety level 4 (BSL-4) containment at the Galveston National Laboratory (GNL), UTMB.

### rVSVΔG-JUNVGP recovery and characterization

A reverse genetics system for the development of rVSVs expressing non-homologous glycoproteins has been described previously^22^. Briefly, the native VSV glycoprotein (G) gene was removed via restriction digest (Mlu1/Nhe1) from a Bluescript plasmid containing the full length VSV genome. A codon-optimized cDNA sequence (GenScript) encoding the JUNV Espindola GPC was ligated in its place. The resulting plasmid was transfected into BHK cells and recovered as described by Lawson et al.^18^. The GPC from JUNV strain Espindola was specifically chosen because this particular virus isolate is considered more virulent in humans and NHP models compared with other prototype strains (e.g., JUNV Romero) and more accurately represents the GPC sequences of currently circulating JUNV strains in Argentina compared with Candid #1 or other attenuated viruses^34,35^. Recovered virus was plaque purified and passaged two times through Vero76 cells. Virus stocks (2 × 10^7^ PFU/ml) were characterized by generating a growth curve in Vero76 cells inoculated at a multiplicity of infection (MOI) of 0.01. Supernatants were harvested every 12 h from 0–96 h post-inoculation (PI). Immunofluorescence assays (IFA) were conducted with rVSVΔG-JUNVGP to evaluate the expression of both the VSV nucleoprotein (N) and the JUNV GP. Vero 76 cells were infected with VSVΔG-JUNV-GPC using a 0.01 multiplicity of infection (MOI). Twenty-four hours post infection (hpi), cells were fixed with 4% paraformaldehyde (Electron Microscopy Science, Hatfield, PA). The fixed monolayers were stained with humanized anti-JUNV-GPC J199 antibody^13^ and mouse anti-VSV-N 10G4 antibody (Kerafast, Boston, MA), followed by staining with goat-anti-human antibody conjugated with Alexa Fluor 488 A-11013 (Thermo Fisher Scientific, Rockford, IL) and/or goat-anti-mouse antibody conjugated with Alexa Fluor 568 A-11031 (Thermo Fisher Scientific, Rockford, IL), and finally counterstaining with 4′,6-diamidino-2-phenylindole (DAPI) (Thermo Fisher Scientific, Rockford, IL). Cellular viral proteins were visualized with an Eclipse Ti fluorescent microscope and images were captured using NIS-Elements software (Nikon, Minato City, Tokyo, Japan). Sequencing was performed by the UTMB Molecular Genomics Core using the Applied Biosciences (ABI) Prism 3130XL DNA sequencer.

### Guinea pig vaccine study

In vivo studies were conducted with outbred female Hartley guinea pigs purchased from Charles River Laboratories. Fifteen animals were divided into prime (P-1 through P-6), prime-boost (B-1 through B-6), and control groups (C-1 through C-3). Three additional historical controls have also been included, where applicable, in our analyses. Animals from the prime and prime-boost groups were vaccinated i.p. with 1 × 10^7^ PFU of rVSVΔG-JUNVGP (day −56). Animals in the prime-boost group received an additional 1 × 10^7^ PFU dose on day −28. Animals (*n* = 6) in the control cohort remained unvaccinated. All animals were challenged i.p. with a uniformly lethal dose (1000 PFU) of JUNV strain Romero (Fig. 1d)^13^. Clinical scores were assessed daily and documented as: Normal (1), Rough (2), Sick (3), Paralysis and/or Euthanize (4). A clinical score of Normal was indicative of a smooth coat and the absence of ocular or nasal discharge. A score of Rough or Sick indicated the presence of either a rough coat and/or ocular discharge; Sick additionally indicated an animal was stationary most of the time but would move when stimulated. Animals were euthanized after scoring Euthanize or Paralysis, when any signs of reluctance or inability to move when stimulated were observed. Weight and temperature measurements were recorded every 3 days between day 0 and 21, day 28, and 35 or terminal for each animal (except for historical controls). Temperatures were assessed via Medic Data Systems, Inc. electronic implantable transponders. Blood was collected via retro-orbital bleed from all animals on the following days: −56, −28, 0, 9, and 35 or terminal (blood was not collected from historical controls on day 9). All animals were necropsied after meeting euthanasia criteria or study endpoint (day 35). Liver, spleen, and brain tissue were collected from all animals upon necropsy. All animal vaccinations were conducted in an ABSL-2 (animal biosafety level 2) facility within the GNL. All subsequent animal work with JUNV was performed in BSL-4 containment at the GNL.

### Animal ethics statement

All animal work was conducted under the approved guidelines of the UTMB Institutional Animal Care and Use Committee (IACUC). Animal research was performed in compliance with the Animal Welfare Act and other Federal statutes and regulations relating to animals and experiments involving animals and adheres to the principles stated in the eighth edition of the *Guide for the Care and Use of Laboratory Animals*, National Research Council, 2011. UTMB is fully accredited by the Association for Assessment and Accreditation of Laboratory Animal Care International.

### Infectious virus

Infectious virus titers in plasma and tissues were determined via plaque assay. Plasma was diluted 10-fold and plated on Vero76 cell monolayers in duplicate wells of six-well plates (200 µl). Plates were incubated at 37^°^ C and rocked every 15 m for 1 h after which 2 ml of 0.8% agarose overlay was plated into each well. Tissues were homogenized into a 10% working dilution (EMEM diluent) using the TissueLyser II (QIAGEN) and titered as above. All plates were stained with 5% neutral red 5 days PI and plaques were read on day 6. The limit of detection for the assay is 25 PFU/ml.

### Antibody titers

IgG antibody titers were assessed via enzyme-linked immunosorbent assay (ELISA). Purified JUNV strain Romero G1/G2 protein as used to coat 96 well polystyrene ELISA plates at 1 µg/ml in 1× PBS. Plasma samples were serially diluted two-fold (starting 1:100 or 1:1000) using 5% BSA/0.05% Tween20 in 1× PBS, plated in triplicate, and incubated overnight at 4 °C. Plates were washed with 0.2% Tween20 in 1xPBS and incubated (1 h) with goat anti-guinea pig IgG conjugated to horseradish peroxidase (HRP) diluted 1:5000 (EMD Millipore Corp.). Plates were washed, incubated with ABTS (2,2′-azino-bis(3-ethylbenzothiazoline-6-sulfonic acid)) peroxidase substrate (KPL) (20–30 m), and read at 405 nm on a Molecular Devices Emax precision microplate reader. Data are reported as IgG reciprocal endpoint dilution titers.

### Antibody avidity

IgG antibody quality was evaluated with an ELISA-based avidity assay using varying concentrations of urea to disrupt antibody/antigen complex binding. ELISA methodologies listed above were modified such that plasma samples for each animal were diluted to achieve a uniform optical density (0.8–1.0). After plasma incubation, plates were washed, 1 M–11 M concentrations of urea (diluted in PBS) were each plated in triplicate, incubated for 15 m at room temperature, and washed before proceeding to secondary antibody incubation. Data are calculated as percent antibody/antigen dissociation compared with PBS-only treated controls.

### Neutralization assays

Plaque reduction neutralization tests (PRNT) were performed to evaluate both the neutralization of rVSVΔG-JUNVGP by JUNV monoclonal antibodies, and to determine neutralizing antibody titers in guinea pig plasma. Plasma was diluted two-fold (1:10 to 1:20,480) in EMEM supplemented with 10% guinea pig complement (Rockland Immunochemicals Inc.)^36^. JUNV monoclonal antibodies (described by^28^) were obtained from Biodefense and Emerging Infections Research Resources Repository (BEI Resources) and diluted 10-fold (10–0.00001 µg/ml) in EMEM. Antibodies GB03-BE08, OD01-AA09, GD01-AG02, and QC03-BF11 are JUNV neutralizing antibodies, while LD05-BF09 and MA03-BE06 are non-neutralizing and target the JUNV GP and JUNV NP, respectively. Plasma and antibody dilutions were incubated 1:1 with 100 PFU of JUNV Espindola, rVSVΔG-JUNVGP, or Candid #1 for 1 h at 37 °C. Plasma/virus dilutions were plated in duplicate (200 µl) on Vero76 cell monolayers with 0.8% agarose overlay. Plates were stained with 5% neutral red and plaques were counted to determine the plaque reduction (PRNT value) for each dilution. All guinea pig plasma samples were normalized to baseline (day −56) plasma samples.

### Histology and immunohistochemistry

Embedded tissues were sectioned, deparaffinized, and antigen retrieval was performed in 10 mM pH6 citrate buffer at 95 °C. Sections were quenched in 3% hydrogen peroxide before being processed for immunohistochemistry (IHC) using the Thermo Scientific Lab Vision Autostainer 360. Avidin D and Biotin (Invitrogen) were applied and sections were treated with Background Buster (Innovex Biosciences) to prevent nonspecific signal. Immunoreactivity was detected using anti-JUNV NP mouse polyclonal 1° antibody at 1:1000 dilution (LifeSpan BioSciences, Inc.), biotinylated goat anti-mouse IgG 2° antibody (Vector Labs) at 1:1600, and streptavidin-HRP (Vector Labs). Slides were developed with Dako DAB chromagen and counterstained with Harris Hematoxylin.

### Statistical analyses

GraphPad Prism 7.03 was utilized for Kaplan–Meier survival curve, unpaired *t*-test (Mann–Whitney), and analysis of variance (ANOVA) statistical analyses of IgG antibody titer, avidity, and neutralization titer. All statistical notations correspond with the following *p* values: *p < 0.05, **p < 0.01, ***p < 0.001.

### Reporting summary

Further information on research design is available in the Nature Research Reporting Summary linked to this article.

## Supplementary information

Reporting Summary

## Data Availability

All datasets used and/or analyzed in the current study are available from the corresponding author upon reasonable request.
